# Assessing the Antecedents and Consequences of Experience Value in Online Education: A Quantitative Approach

**DOI:** 10.3389/fpsyg.2022.911565

**Published:** 2022-06-24

**Authors:** Hong Zhao, Lijuan Song

**Affiliations:** ^1^International College of Cultural Education, Northeast Agricultural University, Harbin, China; ^2^College of Humanities and Social Sciences, East China Jiaotong University, Nanchang, China

**Keywords:** brand image, experience value, continuance intention, affective commitment, online education, marketing strategies

## Abstract

The experience value of online education is a hot topic in both theoretical and practical circles, but research on its mechanism of action is limited. Therefore, this study systematically investigates the relationship between brand image, experience value, and continuance intention through a theoretical analysis of brand image, and discusses the boundary role of effective commitment in it. In this study, 475 users were used to conduct structural equation modeling analysis. The results of the study found that experience value had a positive and significant effect on user continuance intention under the significant influence of brand image, but affective commitment did not play a positive moderating role in the relationship between experience value and continuance intention. This study examines the mechanism of the antecedents and consequences of experience value, and provides a new direction for the construction of online education and the development of online education and marketing strategies.

## Introduction

In the new round of technological revolution, the trend of digitalization and wisdom of industry is becoming more and more obvious, and online education in the virtual environment is no exception (Xiong et al., 2021). From the promotion and application of offline education to the construction of online education, the experience value plays an increasingly important role in the brand building of online education (Rashid et al., 2021). As an important typical representative of virtual communities, online education is driven by the amplification of market demand and the accelerated breakthrough of technological innovation to upgrade the value of user experience, attracting more and more users and showing a broad market prospect (Liu and Qu, 2020; Wang et al., 2021). The instantaneous, interactive, global sharing, low transmission cost, self-management, and open communication characteristics of online education (Sun, 2020; Wang et al., 2020) have made it one of the most popular learning methods in recent years. With the development of information technology, public awareness of the use of online education platforms has sprung up (Ko, 2016; Li et al., 2022). However, given that the growth of users is much slower than the growth of online education platforms, and that many users are concentrated in a few well-known online education platforms, this makes the development of online education more difficult, and competition for users will become increasingly fierce (Li et al., 2021, 2022). The concept of experiential marketing was first introduced by Schmitt (1999), who defined experiential marketing as a process in which individual customers feel certain stimuli after observing or participating in an event, thus inducing motivation and generating thoughtful recognition or purchase behavior. In contrast to traditional marketing, which focuses on product features and price, experiential marketing has a broad content framework that emphasizes the inherent good feelings of consumers regardless of the company, product, brand, or service content (Pine and Gilmore, 1998; Same and Larimo, 2012; Trevinal and Stenger, 2014). Obviously, under the influence of experiential marketing, the public can feel the experiential value of feeling, emotion, and thinking, and this experiential value stimulates users to take a series of actions associated with online education, such as approval or continuance intention.

Studies have shown that experiential value is a series of marketing activities carried out by enterprises through the production and operation of high-quality products, with service products as the platform and tangible products as the carrier, with the goal of meeting consumers' experiential needs and generating feelings and gains (Verhoef et al., 2009; Vada et al., 2020; Bu and Yang, 2021; Prentice et al., 2022). In non-online contexts, the basic concept of experiential value is the value gained from a retail experience through interactions involving direct use or remote viewing (indirect observation) of goods or services, with various cues constantly interacting with customers, not individually, but in an integrated way, providing “value” to customers one fashion retailer at a time (Varshneya and Das, 2017). However, unlike traditional marketing in the past, the value of experience in an online education context is to create a special and unique experience, and consumer psychology and behavior are derived from the experience, so good experience value can bring positive usage psychology and behavior to consumers (Kim, 2012; Yang et al., 2021a). It can be seen that for online education, experiential value has become a new marketing model and represents the future direction of marketing. Implementing experiential value is an inevitable choice for online education companies to brand themselves, build brand loyalty, and construct a competitive advantage (Stavrianea and Kamenidou, 2022). Obviously, for online education, experiential value as a means to enhance the user's perception of the experience and a good reinforcement of the user's continuance intention. As Pine and Gilmore's (1998) study predicted, the era of experiential value has arrived, the marketing battlefield will focus on the creation of great experiences, and the economic form driven by experiential value is the key way to make high profits from online education (Liu et al., 2021; Xing and Li, 2021). Therefore, it is clear that the driving force behind the success of online education operations is the establishment of experiential value. Previous literature has focused on the practical and technical aspects of online education (Shukla, 2012; Chen, 2020). However, there are also studies that suggest that online education is like entertainment media (Hultén, 2012), and that everything is entertainment. Therefore, it will be a challenge for online education managers to master the emotional approach to provide unforgettable experience value to their users and generate the magic that drives users to desire to re-experience (Zhou et al., 2012; Varshneya and Das, 2017).

Existing studies point out that as competition in the online education market continues to intensify, product homogenization is increasing and brand image is increasingly valued by companies and consumers (Kautish and Sharma, 2018). From the point of view of online education users, today's consumers continue to be influenced not only by the product, but also by the brand and its experience value, because this image and its value often represent the user's own image and recognition of the brand value, so online education with a very strong symbolic meaning is more attractive to the public to continuance intention (Varshneya, 2021). From the perspective of online education enterprises, in order to avoid fierce competition in the market, differentiation is often the first choice of enterprises. However, the uniqueness of the brand image is the most reflective of the differences between enterprises and their operations, and this difference in image increases the user's willingness to continue to engage with the brand and is also a competitive advantage that cannot be replicated by other enterprises (Roggeveen et al., 2020; Zhang and Su, 2021). Therefore, online education attaches great importance to brand image shaping and management, which is undoubtedly a wise move, but brand image is rich and there are differences in perspectives, so it is necessary to conduct a systematic study of the mechanism of the role of existing online education brand image (Liu, 2021; Feng and Bao, 2022). To sum up, this study takes online education users as the research object and conducts an empirical study on the relationship between experience value, brand image, and users' continuance intention, to deeply explore and verify the antecedents and consequences of experience value, aiming to understand the mechanism of how experience value and brand image affect users' continuous use of online education, and provide a new direction for the construction of online education and the formulation of online education and marketing strategies.

The remainder of the paper is organized as follows. The next section outlines the theoretical foundation and hypothesis development. After this, the research methodology is explained, followed by the data analysis results. Finally, we present our conclusion.

## Theoretical Foundation and Hypothesis Development

### Brand Image

Since the 1950s, brand image, as an important concept in marketing, has been a hot topic for academics and businesses (Janonis and Virvilaite, 2007; Hyun-Jung, 2013; Huang et al., 2020), and has become a meaningful topic of discussion, commentary, and theoretical construction in the field of business-consumer relations. Brand image is an important component of measuring a brand and needs to be analyzed from different perspectives. As the personality characteristics of a brand expressed in the market and in the minds of the public, brand image reflects consumers' evaluation and perception of the brand (Li et al., 2016). Studies have been conducted based on various elements, such as brand attributes, name, packaging, and reputation, while considering consumers' perceptions of brand image, pointing out that brand reputation and brand personality are important elements reflecting brand image (Li et al., 2016). Brand reputation refers to the brand identity presented by the company, the promises made to consumers, and the extent to which consumers experience the branded product or service, which is a signal sent by the company to the market and is an important representation of brand image (Skard and Thorbjornsen, 2014). Brand personality refers to a set of personality traits associated with a brand, which is important for brand image evaluation. After a brand is given personality traits, it is no longer a passive exchange object, but an active relationship partner. Brands possess personality traits as a natural response from consumers, which can be seen as brand perceptions and feelings formed by consumers in their contact with the brand, reflecting consumers' emotions and embodying their psychological, identity, status, and other personalized needs (Su and Tong, 2015). Undoubtedly, brand image not only reflects the overall impression of a particular brand and represents the message and meaning of the brand; but also represents the personification of the brand and reflects the image of the consumers themselves (Cho et al., 2015).

It is clear that brand image is an important concept in marketing, and this has been agreed upon early on (Gardner and Levy, 1955). Marketing scholars and practitioners agree that the success of products and services is due more to the symbolic meaning of brand image than to physical features and functions (Aaker, 1991). Brand image is also important to the study of brand equity (Keller, 2003). In the article “How Brand Image Drives Brand Equity”, the relationship between brand image and brand equity and the mechanism by which brand image drives brand equity are also clearly presented. Although there is a lot of research on brand image, and with the boom of brand equity research, it has been rapidly increasing. Gardner and Levy (1955), while introducing the concept of branding, criticized past research for being too superficial, focusing only on consumers' stereotypical purchase reasons, and suggested that subsequent research should leave behind superficial purchase reasons and focus on sustainable purchase motivations, i.e., the meaning and value of the brand. However, the meaning and value of brand image in the online education environment has not been clearly verified, and the sustainable purchase motivation of brand image on consumer purchases has yet to be tested. In order to contribute to the study of consumer behavior and to help online education evaluate the effectiveness of brand marketing efforts, this study proposes to apply brand image theory to these new areas of research and to expand its scope of application.

### Brand Image and Experience Value

The earliest research on brand image can be traced back to Gardner and Levy (1955), who argued that brand image is a set of perceptions, feelings, and attitudes that consumers have toward a brand. Subsequently, many scholars from different disciplines (psychology, communication, and management, etc.) have conducted a lot of research on brand image and put forward a series of theories on the concept, model, and essence of brand image. Park et al. (1986) and Biel (1992) have developed different brand concept images (BCM) based on different consumer interests, and measured brand image in terms of functional, symbolic, and experiential markers. The BCM measures the brand image in terms of functional, symbolic and experiential. Biel (1992), believes that brand image is a set of attributes and associations that consumers recall when they see a brand name, including corporate image, product image, and user image. As can be seen, it has become common practice to examine the brand image in users' minds according to three aspects, including functional, symbolic, and experiential markers, in the context of online education usage. In online education, brand image reflects a set of ideas, feelings, and attitudes that the virtual community keeps in users' minds, and the symbolic meaning represented by the brand has a richer connotation and personality (Li et al., 2019). As the emphasis expands from the commodity experience to the brand experience, and with the use of various media to enrich the brand, or to have a connection with the individual's life form, it is the special and memorable consumer experience that is used to differentiate from other brands (Fang et al., 2021). Therefore, a good brand image will increase the value of consumers' experience. Based on this, this article proposes the following hypothesis.

H1: Brand image is positively related to experience value.

### Experience Value, Brand Image, and Continuance Intention

Although academic research on experiential value dates back to 1985, it is believed that the perception of experiential value is primarily derived from consumer interactions with products or services in a direct or distant state, and that these interactions provide the basis for consumer preferences (Holbrook and Corfman, 1985). However, the ability of online education to create and deliver good experiential value to users is increasingly one of the criteria for measuring their core competencies. Experience value can provide both internal and external benefits to consumers (Mano and Oliver, 1993; Babin and William, 1995). Holbrook (1994) added the “activity” dimension to the traditional classification of intrinsic and extrinsic benefits of experiential value, while Mathwick et al. (2001) defined experiential value as the perception of and relative preference for product attributes or service performance. Based on the dimensions proposed by Holbrook (1994), he subdivided experience value into four categories, namely, customer return on investment (CROI), service excellence, aesthetics, and playfulness, and used them as the experiential value scale (EVS). Thus, it can be found that Mathwick et al.'s (2001) EVS goes beyond the traditional focus on the value of combining price and quality, and is able to detect the value components based on experience. Therefore, according to the connotation and essence of Mathwick et al.'s (2001) definition of experiential value, the content of experiential value in online education can be evaluated and measured. That is, in online education environment, experience value reflects the value that results from consumers' sensory, emotional, reflective, action, and associative experiences with online education, including user investment reward, service superiority, aesthetics, and fun.

Research suggests that continuance intention is often a more accurate measure when trying to predict a person's behavior (Engel et al., 1995). Yin et al. (2015) argues that positive continuance intention causes customers to develop a preference for a company and increase the number of purchases of the company's products or services. For the consumer experience of online education, when users have positive continuance intention for the virtual community, they will praise online education, and at the same time, they will develop a preference for using online education, increase the number of times they use online education, and even recommend online education to their friends (Fang et al., 2021). Lial et al. (2019) showed that information value and social value positively influenced consumers' community satisfaction and community identity, which could increase their willingness to participate in brand communities in the future, by collecting data from several brand communities. Interestingly, information value had a greater impact on consumer community satisfaction, but social value had a greater impact on consumer community identification; and community identification had a stronger impact on consumers' willingness to participate in brand communities in the future compared to community satisfaction. A study by Lial et al. (2019) found that online education value is a prerequisite that influences consumers' continued engagement and is the key to the success of online education. Therefore, the value of a good experience will have a positive impact on user continuance intention.

In addition, it has been argued that experience value is an important influencing factor in consumers' evaluation of product continuance intention (Woodruff et al., 1993). More and more companies are understanding that analyzing user satisfaction is an important thing to do, and if managers can identify the factors that influence user satisfaction with a product or service, then the company may be able to change the experience of the consumer in using the product or service to maximize consumer satisfaction (Petrick et al., 2001). In other words, creating good experience value brings consumers greater satisfaction, which leads to positive continuance intention. Aaker (1991) and Rory (2000) also concluded that with a good brand image, consumers would be more satisfied with their purchases and would be willing to recommend them to others, i.e., positive continuance intention. Meng's (2018) study concluded that the brand image of online education has become an important way for companies to expand their customer base and enhance their market competitiveness, and is an important antecedent factor influencing consumption behavior. Therefore, in the online education usage context, a good brand image will have a positive impact on user continuance intention. As a result, this article proposes the hypothesis that:

H2: Experience value is positively related to continuance intention.

H3: Brand image is positively related to continuance intention.

### Moderating Role of Affective Commitment

Affective commitment is the enduring emotional disposition to maintain a valued relationship, and is an essential element for long-term relationship success. It develops on the basis of personal devotion, that is, the individual's desire to maintain a relationship with a long-term perspective of mutual benefit (Bendapudi and Berry, 1997; Yang et al., 2021b). The underlying principle is that affective commitment reflects one's sense of involvement and belonging in the relationship in question. Thus, it encourages the individual to continue the relationship due to the favorable attitudes, influences, feelings, and perceptions held about his or her experience of the relationship. Affective commitment has been considered the most important dimension of commitment to predict continuance intention (Zhou et al., 2015). It has been shown that affective commitment is an important predictor of individual continuance intention in virtual contexts (Malhotra and Galletta, 2005; Li et al., 2010; Zhou et al., 2012). Zhou et al. (2015) investigated the relationship between perceived value, affective commitment, and continuance intention in a virtual socialized world and found that self-indulgence diminishes the effect of utilitarian value and enhances the effect of hedonic value on affective commitment; individualism reduces the effect of relational assets on commitment and weakens the effect of affective commitment on continuance intention.

Experiential value is a core topic of research in the field of relationship marketing. The literature supports the idea that satisfaction, trust, and commitment are the key substructures of continuance intention (Shin et al., 2013). A good relationship between experience value and continuance intention can only be established when users feel valued and make an affective commitment to the operator. It has been shown that experiential value positively influences affective commitment in virtual environments (Kim and Son, 2009; Zhou et al., 2012). In online education, experiential value enhances users' continuance intention to continue with the online education, and the role of experiential value in predicting users' continuance intention is limited due to the uncertainty and risk of online education. Thus, users with higher levels of affective commitment tend to strengthen the advantages of online education and weaken the risks of using online education, thus enhancing the positive effect of experience value on continuance intention. Based on this, and based on the analysis of previous literature, it can be found that affective commitment has a positive moderating effect on the relationship between experiential value and continuance intention. In view of this, the following hypotheses are proposed in this study:

H4: affective commitment plays a positive moderating role between experiential value and continuance intention.

**Figure 1:**
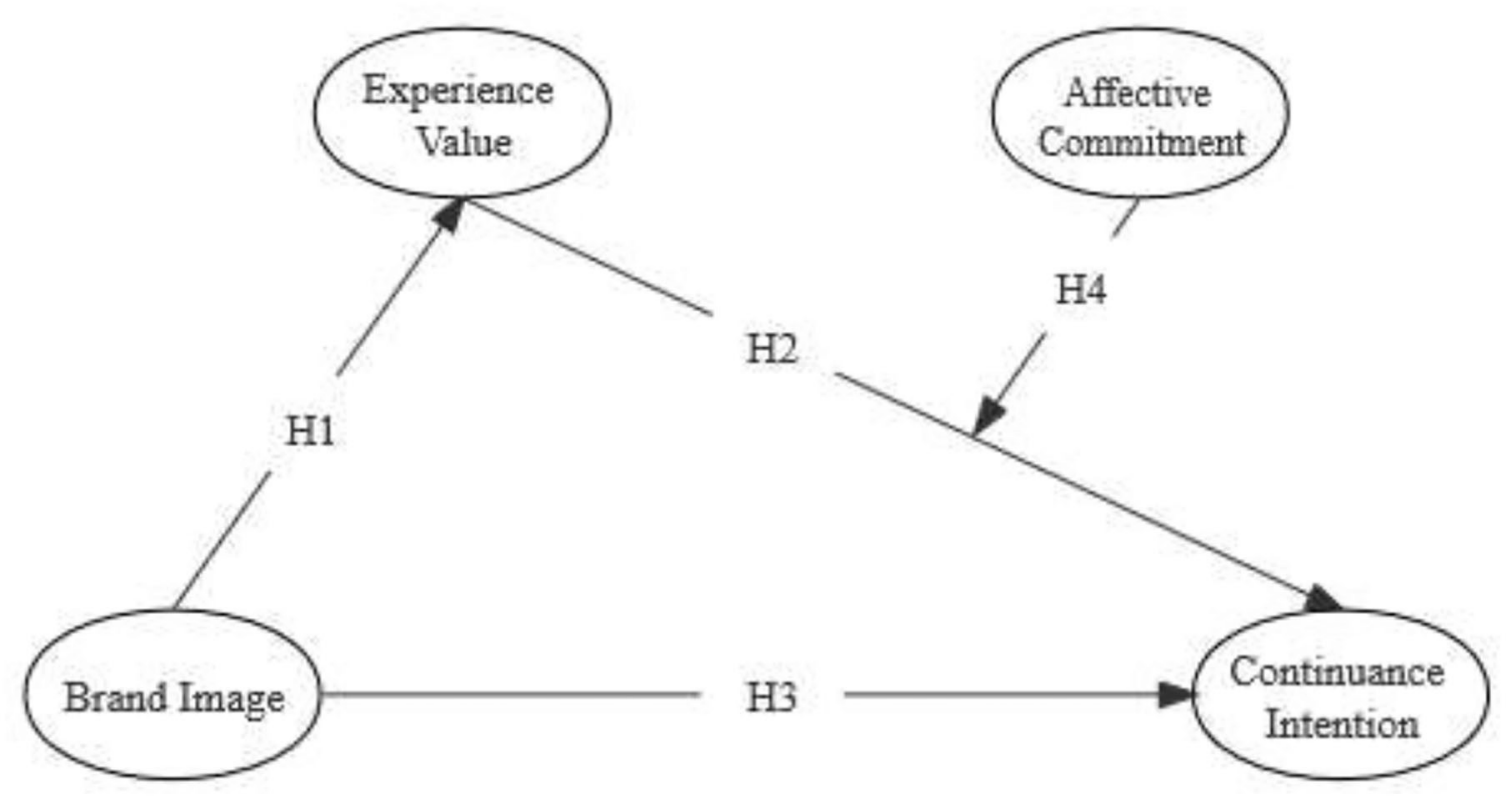
Theoretical model.

In summary, this study proposes a research model as shown in Figure 1.

## Method

### Participants and Measurement Items

In this study, “Thousand Chat” was chosen to collect relevant survey data. In this study, there are two main reasons for using “Thousand Chat” as the survey respondents. The first reason is that “Thousand Chat” has become a mainstream user product as one of the learning tools that users use on a daily basis. In most cases, most people using “Thousand Chat” care not only about the quality of online education, but also other information about the corporate brand of “online education,” and “Thousand Chat” has become an important representative of the brand of online education that users care about. The second reason is that, as one of the largest online education platforms in developing countries, “Thousand Chat” is typical, and if the findings of this study can be supported by data from “Thousand Chat,” it can provide management recommendations for companies in online education to conduct experiential activities. Based on the above analysis, “Thousand Chat” was selected for data collection in this study. The measured constructs and their items are shown in Table 1.

**Table 1:** Variables and measurement item.

**Variables**	**Items**	**Sources**
Brand image	BI1. The Thousand Chat brand has been established.	Sasmita and Mohd, 2015
	BI2. The Thousand Chat has a clear image.	
	BI3. The Thousand Chat has a differentiated image in comparison with the other brand.	
Experience value	EV1. The Thousand Chat has brought me a lot of joy.	Sheth et al., 1991
	EV2. Thousand Chat allows me to find amazing products or special events.	
	EV3. Thousand Chat can make me relax and enjoy myself.	
	EV4. In short, Thousand Chat makes me feel the value.	
Continuance intention	CI1. I share the experience of using TikTok with my friends.	Bhattacherjee, 2001; Thong et al., 2006
	CI2. When I have the need, I use TikTok.	
	CI3. In the future, I will continue to use Tikka.	
	CI4. I recommend TikTok to my friends.	
Affective commitment	AC1. When I use TikTok, I immerse myself unconsciously.	Zhou et al., 2015; Yang et al., 2021b
	AC2. I have a deep affection for TikTok.	
	AC3. TikTok gives me a strong sense of belonging.	
	AC4. TikTok is very attractive to me.	

This paper examines the antecedents and consequences of experiential value in online education. As such, it is a validation study, suitable for data analysis by means of structural equation modeling (SEM). Therefore, it is appropriate for this study to use AMOS 24 to evaluate the empirical data of this paper.

## Data Analysis and Results

### Descriptive Statistical Analysis

In this study, the questionnaire survey was conducted over a period of 5 months from August 2021 to January 2022. In this survey, 600 questionnaires were collected, and 475 valid paired questionnaires were obtained after eliminating invalid questionnaires. The gender distribution in this paper is relatively balanced, with a moderate gender ratio of 50.3 and 49.7%, respectively. In terms of age distribution, the majority of the respondents are young people, and the proportion of respondents aged 30–39 years old is 44.8%. In terms of occupation, the majority of respondents were civil servants (44.2%). In terms of personal monthly disposable income, 5.3% of the respondents had a monthly disposable consumption level of less than RMB 2,000, 14.9% of the respondents had RMB 2,000–3,999, 24.0% of the respondents had RMB 4,000–5,999, and 55.8% of the respondents had RMB 6,000 (including) or more. Respondents generally had an undergraduate education, with only 57 (12.0%) educated to college level or below, 375 (78.9%) educated to undergraduate level, and 43 (9.1%) educated to Master's degree level and above. The details are shown in Table 2. In summary, the sample group in this study has the characteristics of high education, youthfulness, and low personal disposable monthly consumption level, which may be related to the fact that the main target participants of this questionnaire survey are the internet group. In view of this, this study considers that the collected sample data reflects the basic situation of the sample as a whole and is somewhat representative.

**Table 2:** Descriptive statistical analysis.

**Variables**	**Item**	**Frequency**	**%**	**Cumulative %**
Gender	Male	239	50.3	50.3
	Female	236	49.7	100.0
Age (year)	19 or less	9	1.9	1.9
	20–29	187	39.4	41.3
	30–39	213	44.8	86.1
	40–49	47	9.9	96.0
	50 or above	19	4.0	100.0
Marriage	Married	321	67.6	67.6
	Unmarried	151	31.8	99.4
	Divorce	3	0.6	100.0
Profession	Student	47	9.9	9.9
	Freelance	30	6.3	16.2
	Executive in private enterprise	158	33.3	49.5
	Civil servant	210	44.2	93.7
	Clerk in state owned enterprise	24	5.1	98.7
	Executive in private enterprise	6	1.3	100.0
Education	College and blow	57	12.0	12.0
	Undergraduate	375	78.9	90.9
	Master's degree and above	43	9.1	100.0
Consumption (RMB)	Below 2,000	25	5.3	5.3
	2,000–3,999	71	14.9	20.2
	4,000–5,999	114	24.0	44.2
	6,000 or more	265	55.8	100.0
Continuous use time (year)	less than 1	74	15.6	15.6
	1–2	199	41.9	57.5
	Over 3	202	42.5	100.0

### Measurement Model Analysis

The results of the Confirmatory Factor Analysis (CFA) are shown in Table 3. This study evaluates and revises the measurement model of CFA according to the approach of Anderson and Sullivan (1993). That is, CFA should primarily report Cronbach's α, Composite Reliability (CR), and Average Variance Extracted (AVE) for all variables, and only after these metrics pass the test can structural model evaluation be performed (Kline, 2011). Fornell and Lacker (1981), Nunnally and Bernstein (1994), and Hair et al. (2017) clearly stated that when the Cronbach's α is >0.70, the CR is >0.60, and the AVE is >0.50, the measurement model has good convergence validity. In this study, the Cronbach's α is between 0.816 and 0.878, the CR is between 0.824 and 0.882, the AVE is between 0.555 and 0.657. Thus, the results of Cronbach's α, CR and AVE meet the criteria of Fornell and Lacker (1981), Nunnally and Bernstein (1994), and Hair et al. (2017). Therefore, the results of the CFA analysis indicate good convergence validity for all the constructs.

**Table 3:** Confirmatory factor analysis.

**Construct**	**Item**	**Cronbach's α**	**CR**	**AVE**
Brand image (BI)	BI1	0.816	0.824	0.612
	BI2			
	BI3			
Experience value (EV)	EV1	0.832	0.832	0.555
	EV2			
	EV3			
	EV4			
Continuance intention (CI)	CI1	0.878	0.882	0.657
	CI2			
	CI3			
	CI4			
Affective commitment (AC)	AC1	0.848	0.852	0.592
	AC2			
	AC3			
	AC4			

There is discriminant validity when the variance among the constructs of a model is lower than the variance that each construct shares with its items. This study uses the Fornell and Lacker (1981) test to examine the discriminant validity. Table 4 shows that the square root of average variance extracted for each construct is greater than the correlation between the constructs and all other constructs. Therefore, the results of this study support Fornell and Lacker's (1981) recommendation for discriminant validity.

**Table 4:** Discriminant validity for the measurement model.

**Variables**	**Mean**	**SD**	**AVE**	**1**	**2**	**3**	**4**
1. Brand image (BI)	4.654	1.231	0.612	**0.782**			
2. Experience value (EV)	5.012	0.999	0.555	0.436	**0.745**		
3. Continuance intention (CI)	5.756	0.901	0.657	0.354	0.484	**0.811**	
4. Affective commitment (AC)	5.325	1.002	0.592	0.515	0.613	0.619	**0.769**

*The diagonal value is the square root of AVE*.

### Structural Model Analysis

Model fit was analyzed according to the recommendations of Jackson et al. (2009) in this study. The results found ML χ^2^ = 48.564, DF = 41, 1 < Normed Chi-sqr (χ^2^/DF) = 1.184 < 3, RMSEA = 0.020 < 0.08, SRMR = 0.021 < 0.08, TLI (NNFI) = 0.996 > 0.9, CFI = 0.997 > 0.9, GFI = 0.982 > 0.9, AGFI = 0.955 > 0.9. This indicates that the structural model of this study has a good fit (Bollen and Stine, 1992). The path coefficients are shown in Table 5. Brand image (BI) (β = 0.275, *p*-value < 0.001) is positively associated with experience value (EV). Therefore, H1 is supported. Experience value (EV) (β = 0.397, *p*-value < 0.001) is positively associated with continuance intention (CI). Therefore, H2 is accepted. Brand image (BI) (β = 0.108, *p*-value < 0.01) is positively associated with continuance intention (CI). Therefore, H3 is also accepted.

**Table 5:** Regression coefficient.

	**Unstd**	**S.E**.	**Unstd./S.E**.	**Std**.	***p*-value**
H1: BI->EV	0.275	0.037	7.521	0.435	***
H2: EV->CI	0.397	0.059	6.670	0.407	***
H3: BI->CI	0.108	0.033	3.237	0.175	**

*BI, Brand Image; EV, Experience Value; AC, Affective Commitment; CI, Continuance Intention.*

** *p-value < 0.01,*

*** *p-value < 0.001*.

The moderating effects are reported in Table 6. In the present study, affective commitment (AC) is the moderating variable. The results of structural equation modeling have been shown that the moderator effect of experience value (EV) × affective commitment (AC) on continuance intention (CI) is −0.034 (z = |−1.015| < 1.96, *p*-value > 0.001), implying the presence of a positive moderating effect of affective commitment (AC) on the relationship between experience value (EV) and continuance intention (CI). Specifically, the slope of experience value (EV) on continuance intention (CI) increases negatively by −0.034 units for each 1-unit increase in the moderating variable affective commitment (AC). That is, experience value (EV) has a negative moderating effect. Therefore, hypothesis 4 is not verified.

**Table 6:** The analysis of moderating effect.

**DV**	**IV**	**Path coefficient (β)**	**S.E**.	**Z-value**	***p*-value**
Continuance intention (CI)	Experience value (EV)	0.187	0.052	3.569	***
	Affective commitment (AC)	0.464	0.063	7.418	***
	Experience value (EV) × Affective commitment (AC)	−0.034	0.034	−1.015	ns

*** *p < 0.001; ns, non-significant*.

## Research Results and Discussion

### Conclusions

First, the results of the study indicate that brand image has a positive and significant impact on continuance intention. The findings are consistent with the conclusions drawn from Meng's (2018) study. This is because online education is full of widespread and distorted information, which makes online education users cautious when they experience the fun during online education with their senses, thoughts, and feelings, and they do not know whether the online education is trustworthy or not. But a good experience will leave a good image of that online education provider in the user's mind, and multiple good experiences keep strengthening this positive image, thus making the online education users feel more and more satisfied with the use of that online education provider. Therefore, companies should focus on strengthening the brand image of online education, establishing an honest and reliable image in the virtual world where online education is plagued by viruses and flooded with false information, and should work to replace viral online education methods with high-quality information services and fast response speed.

Second, there is a positive and significant impact of the brand image of online education on the value of the experience. The findings are consistent with the Fang et al. (2021) study, which may be related to the fact that online education security is the main key feature, and the security performance of the online education as well as functional features, product prices, and other functional images will directly affect the brand of the online education, which are elements valued by the users of online education, so the brand image plays an role in online education. When such users are attracted by the practicality and artistry of the online education content, the online education provider will secure users thanks to the terms of information and service performance. In addition, for online education, the role of brand image in influencing user behavior is more pronounced. This is because online education tends to speak from “strength” and use the quality of data to attract online education users. Therefore, brand image plays a relatively important role in influencing users' continuance intention.

Third, the findings suggest that experiential value has a positive and significant effect on continuance intention. The findings are consistent with the conclusions reached by scholars such as Lial et al. (2019) in their previous studies. It is speculated that the reason may be because both the experiential marketing of online education and their brand image positively influence users' continuance intention through the mediator of users' experience value. Therefore, in the management practice of online education, it is not enough to implement experiential marketing and branding strategies, but it is also necessary to introduce more relevant tools through management marketing innovation to enhance users' experience value, so that users of online education will visit again and recommend the website to their friends and family. Therefore, when building online education, companies should pay attention to the overall aesthetics of the online education page, and the placement of advertisements should be integrated within the entire online education platform.

Fourth, the results of the study showed that affective commitment did not have a positive moderating effect on the effect of experience value on continuance intention. According to the results of the moderating effect analysis, it is clear that affective commitment does not have a positive moderating effect between experiential value and intention to use. Users with higher affective commitment tend to be more emotionally attached to online education operators based on long-term interaction. Users with higher affective commitment tend to trust service providers more, see the advantages of online education more easily, and tolerate certain inappropriate behavior from online education operators; thus their experience value has a stronger impact on continuance intention. On the contrary, users with lower affective commitment lacked trust in the online education service provider and interacted less with the online education service provider. Therefore, users with a higher experience value may be less willing to use online education than those with higher levels of affective commitment.

### Theoretical Contributions

On the one hand, this paper breaks through the limitations of previous studies, which focused on the influence of motivation and behavior on the behavior of users in online education, and starts from the perspective of the online education experience, focusing on the psychological perception of members, while considering the antecedents and consequences of experience value. The theoretical model of the influence of community members' experience on their continuance intention is constructed based on the literature on brand image. This model has contributed to the development of the theory and practice of online education.

On the other, the rise of the internet has diversified the communication channels between companies and users, and the establishment of online education is a new form of interactive communication between companies and users, and therefore has received increasing attention from academics. However, the existing theories on online education mainly focus on the impact on brand loyalty from the way community members participate in communication, but rarely focus on the community experience that plays an important role in users' psychological perception. This paper examines users' experience value and its antecedents and consequences through an empirical study to validate the overall theoretical model, thus broadening the research channel on the experience value of online education and extending the study of experience value to the field of online education.

### Practical Implications

First, for online education, the role of experiential value is to gain insight into users' motivations and needs for participation, and to design policies that reinforce users' sense of commitment and participation in the community. This paper shows that experiential value is a key psychological link between users and communities, which provides guidance to community operators on how to strengthen the design of user “identity” display mechanisms in online education. In addition to creating a positive brand image, it is also important to actively encourage users' own perceptions of the community experience. Online education operators need to give users adequate tools for self-expression, such as reputation, ranking, rating systems, signatures, and personal websites, and online education providers need to take full advantage of technology-mediated features to support the construction of users' experience needs and the experiential interactions that occur through identity presentation.

Second, improving the brand image of online education is a management component that community operators need to focus on. Offline connections in online education only occur when a brand image is generated within the community, and offline connections among online education members are increasingly important to the cohesiveness of online education. Of course, increasing interaction is a means to an end, but so is promoting a social identity within online education. This can be seen from two perspectives. One perspective is that, at the technical level, functional modules can be developed and designed to promote brand image management, such as the cross-group, live-streaming function of the online knowledge community represented by the Thousand Chat community; another perspective is that at the management level, operators of online education can set up dedicated interpersonal managers to regularly and purposefully guide mutual communication and sharing among members in the community, such as fan groups, regular offline parties and promotional activities, so that brand image promotion becomes a normal phenomenon in online education.

Third, online education managers can influence users' continuance intention through the policy control of brand image, and enhance the experience to bring value to users through various technical features of online education, real-time communication, interactive content (UGC), and various interactive tools application, etc., in order to promote the realization of individual consumption psychology and behavior. Therefore, in order to guarantee the effectiveness of experiential marketing, the community can regularly evaluate and monitor both the form and level of experiential marketing as well as the effectiveness of experiential marketing. In addition, different virtual community design policies can be adopted. For example, when developing communities that focus on interpersonal connections the formation of relationships between members of the community must be promoted. This means that it is not enough to have online interactions; offline activities between members should also be encouraged. Accordingly, this requires community policies that encourage more direct sharing of private information between members.

Fourth, it is important to categorize and manage online education users. In online education, empirical results show that users with high levels of affective commitment are more important for brand image, value experience, and continuance intention, which means that community operators should have a very high level of content creation so that they can guide, control, and create new content to retain the group of users who stay in the wider community. Therefore, before developing and implementing marketing initiatives, online education providers first need to segment and target user groups in the market, manage the categories, and focus their efforts on key users. For example, users who use online education more than once a week have higher brand image, experience value, and willingness to use the online education than other frequent users. For this group, online education needs to emphasize the innovative points of online education in the marketing process, and enhance the value of user experience through differentiated marketing activities.

### Limitations and Future Research

Although this paper draws some conclusions about the importance of both theory and practice of online education experience marketing, there are still some limitations. Since users of different genders, geographic regions, and consumption levels can have differences in online literacy, this can affect their feelings about the online education experience; however, this paper does not classify the sample by the above characteristics for empirical study. Subsequent research could compare the differences in gender, geography, and consumption inequality characteristics to find out the impact of these characteristics on the relationship chain in this paper.

## Data Availability Statement

The original contributions presented in the study are included in the article/supplementary material, further inquiries can be directed to the corresponding author.

## Ethics Statement

Ethical review and approval was not required for the study on human participants in accordance with the local legislation and institutional requirements. Informed consent was obtained from all subjects involved in the study.

## Author Contributions

HZ: conceptualization and writing original draft. LS: formal analysis and investigation. HZ and LS: writing—review and editing. All authors have read and agreed to the published version of the manuscript.

## Conflict of Interest

The authors declare that the research was conducted in the absence of any commercial or financial relationships that could be construed as a potential conflict of interest.

## Publisher's Note

All claims expressed in this article are solely those of the authors and do not necessarily represent those of their affiliated organizations, or those of the publisher, the editors and the reviewers. Any product that may be evaluated in this article, or claim that may be made by its manufacturer, is not guaranteed or endorsed by the publisher.

## References

[B1] Aaker D. (1991). Manage Brand Equity. New York, NY: The Free Press.

[B2] Anderson E. W. Sullivan M. (1993). The antecedents and consequences of customer satisfaction for firms. Mark. Sci. 12, 125–143. 10.1287/mksc.12.2.125

[B3] Babin B. J. William R. (1995). Consumer self - regulation in a retail environment. J. Retail. 71, 47–70. 10.1016/0022-4359(95)90012-8

[B4] Bendapudi N. Berry L. L. (1997). Customers' motivations for maintaining relationships with service providers. J. Retail. 73, 15–37. 10.1016/S0022-4359(97)90013-0

[B5] Bhattacherjee A. (2001). Understanding information systems continuance: an expectation-confirmation model. MIS Q. 25, 351–370. 10.2307/3250921

[B6] Biel A. (1992). How brand image drives brand equity. J. Advert. Res. 32, 6–12.

[B7] Bollen K. A. Stine R. A. (1992). Bootstrapping goodness-of-fit measures in structural equation models. Sociol. Methods Res. 21, 205–229. 10.1177/0049124192021002004

[B8] Bu C. L. Yang F. (2021). YY: A new form of online education. Modern Educ. Technol. 24, 94–100. 10.3969/j.issn.1009-8097.2014.09.013

[B9] Chen X. H. (2020). The call of the times for constructing online education theory. China Educ. Technol. 8, 22–26. 10.3969/j.issn.1006-9860.2020.08.004

[B10] Cho E. Fiore A. M. Russell D. W. (2015). Validation of a fashion brand image scale capturing cognitive, sensory, and affective associations: testing its role in an extended brand equity model. Psychol. Market. 32, 28–48. 10.1002/mar.20762

[B11] Engel J. F. Blackwell R. D. Miniard P. W. (1995). Consumer Behavior. Chicago, New York: Dryden Press.

[B12] Fang X. M. Chen Y. Zhu L. L. (2021). Knowledge mapping analysis of online education Research in China Based on CiteSpace. J. Bengbu Univ. 10, 44–49. 10.3969/j.issn.2095-297X.2021.01.011

[B13] Feng J. Q. Bao H. (2022). The dilemma of private book industry K12 online education investment and countermeasures. View Publish. 3, 48–52. 10.16491/j.cnki.cn45-1216/g2.2022.03.009

[B14] Fornell C. R. Lacker D. F. (1981). Structural equation models with unobservable variables and measurement error. J. Mark. Res. 18, 382–388. 10.1177/002224378101800313

[B15] Gardner B. G. Levy S. J. (1955). The product and the brand. Harv. Bus. Rev. 33, 33–39.

[B16] Hair J. F. Hult G. T. M. Ringle C. M. Sarstedt M. Thiele K. O. (2017). Mirror, mirror on the wall: a comparative evaluation of composite-based structural equation modeling methods. J. Acad. Mark. Sci. 45, 616–632. 10.1007/s11747-017-0517-x

[B17] Holbrook M. B. (1994). The Nature of Customer Value: An Axiology of Service in the Consumption Experience, Service Quality: New Direction in Theory and Practice. Thousand Oaks, CA: Sage.

[B18] Holbrook M. B. Corfman K. P. (1985). Perceived Quality: How Consumers View Stores and Merchandise. Lexington, MA: Lexington Books, 31–57.

[B19] Huang L. Wang M. Chen Z. Deng B. Huang W. (2020). Brand image and customer loyalty: transmitting roles of cognitive and affective brand trust. Soc. Behav. Person. Int. J. 48, 1–12. 10.2224/sbp.9069

[B20] Hultén B. (2012). Sensory cues and shoppers' touching behaviour: the case of IKEA. Int. J. Retail Distrib. Manage. 40, 273–289. 10.1108/09590551211211774

[B21] Hyun-Jung L. (2013). How consistency of brand image and advertising image for parent and extended brands affects brand attitude. Res. J. Costume Cult. 21, 546–561. 10.29049/rjcc.2013.21.4.546

[B22] Jackson D. L. Gillaspy J. A. Purc-Stephenson R. (2009). Reporting practices in confirmatory factor analysis: an overview and some recommendations. Psychol. Methods. 14, 6–23. 10.1037/a0014694 19271845

[B23] Janonis V. Virvilaite R. (2007). Brand image formation. Eng. Econ. 52, 78–90.

[B24] Kautish P. Sharma R. (2018). Consumer values, fashion consciousness and behavioral intentions in the online fashion retail sector. Int. J. Retail Distrib. Manage. 46, 894–914. 10.1108/IJRDM-03-2018-0060

[B25] Keller K. L. (2003). Brand synthesis: The multidimensionality of brand knowledge. J. Consum. Res. 29, 595–600. 10.1086/346254

[B26] Kim H. (2012). The dimensionality of fashion-brand experience: aligning consumer-based brand equity approach. J. Fashion Mark. Manage. 16, 418–441. 10.1108/13612021211265827

[B27] Kim S. S. Son J. Y. (2009). Out of dedication or constraint? A dual model of post-adoption phenomena and its empirical test in the context of online services. MIS Q. 33, 49–70. 10.2307/20650278

[B28] Kline R. B. (2011). Principles and Practice of Structural Equation Modeling. 3th Edn. New York: Guilford.

[B29] Ko T. T. (2016). Rethinking and strategies for international online education. Heilongjiang Res. High. Educ. 10, 91–93. 10.3969/j.issn.1003-2614.2016.10.024

[B30] Li D. Browne G. J. Chau P. Y. K. (2010). An empirical investigation of web site use using a commitment based model. Decis. Sci. 37, 427–444. 10.1111/j.1540-5414.2006.00133.x

[B31] Li M. Hua Y. Zhu J. X. (2021). From interactivity to brand preference: the role of social comparison and perceived value in a virtual brand community. Sustainability 13, 625. 10.3390/su13020625

[B32] Li X. Zhang M. L. Luo N. (2016). The impact of brand image on brand relationship benefits. J. Manage. Sci. 29, 120–130. 10.3969/j.issn.1002-3291.2019.04.008

[B33] Li X. X. Guo C. Yu T. (2019). Effects of the interactions of brand virtual community on consumer brand evangelism. J. Liaoning Univ. 47, 47–54.

[B34] Li Y. L. Wang Y. L. Feng X. L. Li X. R. (2022). An analysis of changes in the development of online education in the context of 5G era. Modern Vocat. Educ. 3, 34–36.

[B35] Lial J. Y. Lin X. X. Wei H. Y. (2019). How does online brand community value influence consumer's continuous participation? The moderating role of brand knowledge. Nankai Bus. Rev. 6, 16–26. 10.3969/j.issn.1008-3448.2019.06.003

[B36] Liu L. B. Qu M. X. (2020). A perspective on online education supported by information technology. Modern Educ. Manage. 108–114. 10.16697/j.1674-5485.2020.08.015

[B37] Liu W. (2021). Implications of Internet thinking for online education. Video Eng. 45, 154–156. 10.16280/j.videoe.2021.06.044

[B38] Liu G. P. Wang X. Gao N. Hu H. L. (2021). From virtual reality to metaverse: new directions for online education. Modern Distance Educ. Res. 33, 12–22.

[B39] Malhotra Y. Galletta D. (2005). A multidimensional commitment model of volitional systems adoption and usage behaviour. J. Manage. Inform. Syst. 22, 117–151. 10.1080/07421222.2003.11045840

[B40] Mano H. Oliver R. L. (1993). Assessing the dimensionality and structure of the consumption experience: evaluation, felling and satisfaction. J. Consum. Res. 20, 451–466. 10.1086/209361

[B41] Mathwick C. Malhotra N. Rigdon E. (2001). Experiential value: conceptualization, measurement and application in the catalog and internet shopping environment. J. Retail. 77, 39–56. 10.1016/S0022-4359(00)00045-2

[B42] Meng L. (2018). The influence of perceived value of virtual brand communities on consumer behaviour. Business Econ. Res. 2, 46–49. 10.3969/j.issn.1002-5863.2018.02.01435602701

[B43] Nunnally J. C. Bernstein I. H. (1994). Psychometric Theory. New York, NY: McGraw-Hill.

[B44] Park C. W. Joworski B. J. Machinnis D. J. (1986). Strategic brand concept - image management. J. Mark. 50, 135–145. 10.1177/002224298605000401

[B45] Petrick J. F. Morais D. D. Norman W. C. (2001). An examination of the determinants of entertainment vacationer's intentions to revisit. J. Travel Res. 40, 41–48. 10.1177/004728750104000106

[B46] Pine B. J. Gilmore J. H. (1998). Welcome to the experience economy. Harv. Bus. Rev. 76, 97–105. 10181589

[B47] Prentice C. Dominique-Ferreira S. Ferreira A. Wang X. Q. (2022). The role of memorable experience and emotional intelligence in senior customer loyalty to geriatric hotels. J. Retail. Consum. Serv. 64, 102788. 10.1016/j.jretconser.2021.102788

[B48] Rashid N. Nika F. A. Thomas G. (2021). Impact of service encounter elements on experiential value and customer loyalty: an empirical investigation in the coffee shop context. Sage Open. 11, 21582440211061385. 10.1177/21582440211061385

[B49] Roggeveen A. L. Grewal D. Schweiger E. B. (2020). The DAST framework for retail atmospherics: the impact of in-and out-of-store retail journey touchpoints on the customer experience. J. Retail. 96, 128–137. 10.1016/j.jretai.2019.11.002

[B50] Rory P. M. (2000). A consumer-orientated framework of brand equity and loyalty. Int. J. Market Res. 42, 65–78. 10.1177/147078530004200105

[B51] Same S. Larimo J. (2012). Marketing theory: experience marketing and experiential marketing, in 7th International Scientific Conference on Business and Management, 480–487.

[B52] Sasmita J. Mohd S. N. (2015). Young consumers' insights on brand equity. Int. J. Retail Distrib. Manage. 43, 276–292. 10.1108/IJRDM-02-2014-0024

[B53] Schmitt B. (1999). Experiential Marketing: How to Get Customers to Sense Feel, Think, Act, Relate to Your Company and Brands. Simon and Schuster Inc.

[B54] Sheth J. N. Newman B. I. Gross R. B. L. (1991). Why we buy what we buy: a theory of consumption values. J. Bus. Res. 22, 159–170. 10.1016/0148-2963(91)90050-8

[B55] Shin J. Chung K. Oh J. Lee C. (2013). The effect of site quality on repurchase intention in Internet shopping through mediating variables: the case of university students in South Korea. Int. J. Inf. Manage. 33, 453–463 10.1016/j.ijinfomgt.2013.02.003

[B56] Shukla P. (2012). The influence of value perceptions on luxury purchase intentions in developed and emerging markets. Int. Mark. Rev. 29, 574–596. 10.1108/02651331211277955

[B57] Skard S. Thorbjornsen H. (2014). Is publicity always better than advertising? The role of brand reputation in communicating corporate social responsibility. J. Bus. Ethics 124, 149–160. 10.1007/s10551-013-1863-3

[B58] Stavrianea A. Kamenidou I. (2022). Complying with digital transformation in online booking through experiential values of generation Z. Eur. J. Tour. Res. 30, 3003–3003. 10.54055/ejtr.v30i.2590

[B59] Su J. Tong X. (2015). Brand personality and brand equity: evidence from the sportswear industry. J. Product Brand Manage. 24, 124–133. 10.1108/JPBM-01-2014-0482

[B60] Sun C. (2020). Evaluation of the consumer interaction capability of the virtual brand community of Chinese mobile internet enterprises based on the hybrid MCDM method. J. Inform. Knowledge Manage. 19, 2050034. 10.1142/S0219649220500343

[B61] Thong J. Y. L. Hong S. J. Tam K. Y. (2006). The effects of post-adoption beliefs on the expectation-confirmation model for information technology continuance. Hum. Comp. Stud. 64, 799–810. 10.1016/j.ijhcs.2006.05.001

[B62] Trevinal A. M. Stenger T. (2014). Toward a conceptualization of the online shopping experience. J. Retail. Cons. Serv. 21, 314–326.

[B63] Vada S. Prentice C. Scott N. Hsiao A. (2020). Positive psychology and tourist well-being: a systematic literature review. Tour. Manage. Perspect. 33, 100631. 10.1016/j.tmp.2019.100631

[B64] Varshneya G. (2021). Antecedents and consequences of experiential value in fashion retailing: a study on Indian consumers. J. Fashion Mark. Manage. 10.1108/JFMM-06-2020-0113

[B65] Varshneya G. Das G. (2017). Experiential value: multi-item scale development and validation. J. Retail. Consum. Serv. 34, 48–57. 10.1016/j.jretconser.2016.09.010

[B66] Verhoef P. C. Lemon K. N. Parasuraman A. Roggeveen A. Tsiros M. Schlesinger L. A. (2009). Customer experience creation: determinants, dynamics and management strategies. J. Retail. 85, 31–41. 10.1016/j.jretai.2008.11.001

[B67] Wang L. Sakashita M. Cheng G. P. Ji J. Z. Zhang Y. T. (2020). The effect of regulatory focus on customer citizenship behavior in a virtual brand community: the role of online self-presentationand community identification. J. Consum. Behav. 20, 607–625. 10.1002/cb.1888

[B68] Wang J. Zheng H. Li W. (2021). Online education governance in the age of intelligence: connotations, dilemmas and breakthroughs. E Educ. Res. 42, 54–60. 10.13811/j.cnki.eer.2021.07.008

[B69] Woodruff R. B. Schumann D. W. Gardial S. F. (1993). Understanding value and satisfaction from the customer's point of view. Survey Bus. 29, 33–40. 26903927

[B70] Xing X. S. Li J. (2021). New ideas for the development of online education in the era of “Internet+”. China Educ. Technol. 5, 57–62. 10.3969/j.issn.1006-9860.2021.05.008

[B71] Xiong T. C. Zhao Y. Y. Xu L. Liu D. L. (2021). Research on online education platform development. Cooperat. Econ. Sci. 11, 61–65. 10.3969/j.issn.1672-190X.2021.11.026

[B72] Yang M. Hu S. Kpandika B. E. Liu L. (2021b). Effects of social attachment on social media continuous usage intention: the mediating role of affective commitment. Hum. Syst. Manage. 40, 619–631. 10.3233/HSM-201057

[B73] Yang M. S. Zhang W. S. Ruangkanjanases A. Zhang Y. (2021a). Understanding the mechanism of social attachment role in social media: a qualitative analysis. Front. Psychol. 12, 720880. 10.3389/fpsyg.2021.720880 34421773 PMC8378210

[B74] Yin F. S. Liu M. L. Lin C. P. (2015). Forecasting the continuance intention of social networking sites: assessing privacy risk and usefulness of technology. Technol. Forecast. Soc. Change 99, 267–272. 10.1016/j.techfore.2015.07.019

[B75] Zhang J. W. Su H. L. (2021). Knowledge extension and existential contraction of remote presence: an existential interpretation of online education. Educ. Res. 27, 65–70. 10.13966/j.cnki.kfjyyj.2021.01.007

[B76] Zhou Z. Fang Y. Vogel D. Jin X. L. Zhang X. (2012). Attracted to or locked in? Predicting continuance intention in social virtual world services. J. Manage. Inform. Syst. 29, 273–306. 10.2753/MIS0742-1222290108

[B77] Zhou Z. Jin X. L. Fang Y. Vogel D. (2015). Toward a theory of perceived benefits, affective commitment, and continuance intention in social virtual worlds: cultural values (indulgence and individualism) matter. Eur. J. Inform. Syst. 24, 247–261. 10.1057/ejis.2014.27

